# Acquired hemophilia A in a patient treated by Ginkgo‐dipyridamolum

**DOI:** 10.1002/ccr3.712

**Published:** 2017-01-14

**Authors:** Jinbo Liu, Hongyu Wang, Guangyun Shang, Lichun Wang, Hongwei Zhao, Huan Liu

**Affiliations:** ^1^ Department of Vascular MedicinePeking University Shougang HospitalBeijingChina

**Keywords:** Acquired hemophilia A, Ginkgo‐dipyridamolum

## Abstract

Acquired hemophilia A might be caused by Ginkgo‐dipyridamolum especially by Ginkgo, and it was successfully treated with hemostasis and immune‐suppression therapy including methylprednisolone and cyclophosphamide.

## Introduction

We reported a case of 84‐year‐old women treated with Ginkgo‐dipyridamolum because of thrombus of peroneal vein. She was diagnosed with acquired hemophilia A for high‐titer factor VIII (FVIII) inhibitor and lower level of FVIII activity. aPTT returned to normal range after treated with corticosteroids and cyclophosphamide.

Acquired hemophilia A (AHA) is a bleeding disorder caused by an autoantibody to factor VIII (FVIII), presenting with serious and fatal bleeding such as subcutaneous and gastrointestinal bleeding 1. Therapy of bleeding control and immune suppression must be given immediately to save patient's life 2. Patients with bleeding and prolonged level of activated partial thromboplastin time (aPTT) with normal level of bleeding time, prothrombin time, and thrombin time should be considered as this disease. Evidence of higher level of FVIII inhibitor titer and lower level of FVIII activity will confirm the diagnosis of this disease 3. Ginkgo‐dipyridamolum is used for the treatment of venous thromboembolism approved by China Food and Drug Administration. Here, we reported a rare case of AHA might be related with Ginkgo‐dipyridamolum, and it was successfully treated with hemostasis and immune‐suppression therapy.

## Case Presentation

A patient (women, 84 years old) visited Peking University Shougang Hospital with swelling and pain in the right leg for 2 days on 6 May 2015. Her medical history was coronary artery disease confirmed by coronary angiography (stenosis of anterior and posterior descending coronary artery (60% and 80%, respectively), occlusion of middle part of left circumflex artery), hypertension and hyperlipidemia, without history of stroke, diabetes mellitus, peripheral artery disease, autoimmune diseases, and tumor diseases. Her usual treatment included aspirin 100 mg/day, clopidogrel 75 mg/day, metoprolol succinate sustained release 71.25 mg/day, atorvastatin 10 mg/day, and amlodipine besylate 5 mg/day. All of these drugs had been used for 1 year. In addition, she was treated with low‐molecular weight heparin in July 2013 because of unstable angina without abnormality of coagulation function. There were no family histories of bleeding disorders and other hemophilia‐related diseases. Another medical history was that she had pain and swelling in her right knee for some days after leg pressing.

Whole‐blood count analysis revealed a white blood cell count of 7.5 × 10^9^/L (reference range 3.5–9.5 × 10^9^/L) with normal differentials, hemoglobin level of 119 g/L (reference range 115–150 g/L), and a platelet count of 151 × 10^9^/L (reference range 125–350 × 10^9^/L). The result of coagulation examination showed an aPTT of 20 sec (reference range 24–35 sec) with normal bleeding time, prothrombin time, and thrombin time. The results of electrolytes, urea, creatine, and liver function tests were all normal. Ultrasound showed thrombus of peroneal vein in the right leg. Low‐molecular weight heparin 3200 iu/day 4 and Ginkgo‐dipyridamolum 20 mL (Ginkgo 22.0 mg, and dipyridamolum 8.8 mg)/day were initiated, and aspirin and clopidogrel were discontinued.

The orthopedic doctor did the joint cavity puncture on the right knee and extracted dark red liquid (about 100 mL) on 7 May 2015. This manifestation suggested there was some bleeding in the cavity, and low‐molecular weight heparin and Ginkgo‐dipyridamolum were held immediately. However, aPTT was prolonged to 126.6 sec without abnormality in bleeding time, prothrombin time, and thrombin time on the next morning. The value of aPTT was higher in the next examination on afternoon 8 May 2015 (more than 180 sec) with lower hemoglobin (70 g/L). However, there was no abnormality on the results of electrolytes, urea, creatine, liver function, and fecal occult blood tests. Physical examination showed that there were large hematomas on the right upper arm, axillary, and breast. We gave blood plasma 200 mL Qd, red blood cell suspension 2 u Qd (for 4 days), protamine sulfate injection 50 mg/day (for 2 days), and human prothrombin complex 300 iu bid (for 2 days) for symptom treatment. But the value of aPTT was still high (more than 120 sec) with normal bleeding time, prothrombin time, thrombin time, and stable hemoglobin (about 90 g/L).

Further examination showed that tumor markers, lung CT, and abdominal ultrasound were normal. Thromboelastogram showed that there was no heparin effect. Coagulation tests showed markedly prolonged aPTT (129.5 sec), which was partially corrected on an immediate 1:1 mixing study with normal plasma (25.2 sec). However, the mixing test results by prolonged incubation for 2 h at 37°C showed prolongation (120.2 sec), not corrected on a 2‐h 1:1 mixing study with normal plasma. Further laboratory tests showed that FVIII activity was 0.1% (reference range 50–150%), with normal level of FXII activity (77.4%, reference range 50–150%), normal level of FIX activity (112.7%, reference range 65–150%), decreased level of FXI activity (32.7%, reference range 65–150%). FVIII inhibitor was 20.8 Bethesda units (BU) (reference range <0.6). The result of lupus anticoagulant (LA) test was 1.23 (reference range ≤1.2). Other autoantibodies including anticardiolipin antibody, antinuclear antibody, double‐stranded DNA antibody, rheumatoid factor, and von Willebrand factor were negative. She was diagnosed with AHA.

According to the diagnosis and recommendation of AHA, she was treated with corticosteroids (methylprednisolone 60 mg intravenously/day), cyclophosphamide (400 mg twice a week). In addition, calcium and vitamin D were used to prevent osteoporosis. The level of aPTT decreased to 49.5 sec after 3 weeks. The treatment was changed to corticosteroids (methylprednisolone 40 mg intravenously/day), cyclophosphamide (200 mg, three times a week). aPTT returned to the normal range with normal level of electrolytes, urea, creatine, and liver function, and there were no further hemorrhagic manifestations after 4 weeks. Factor assays showed that the level of FVIII activity was 24.6%, and Bethesda assays showed a low‐titer FVIII inhibitor at 0.5 BU, both obviously improved than before. After 6 weeks, the level of FVIII activity was 138.5%, and the level of FVIII inhibitor was 0.0 BU, completely restored to normal.

This patient was treated by aspirin 100 mg/day, clopidogrel 75 mg/day, metoprolol succinate sustained release 71.25 mg/day, atorvastatin 10 mg/day, and amlodipine besylate 5 mg/day because of coronary artery disease, hypertension, and hyperlipidemia after hospital discharge on 5 August 2015. And aPTT was 33.9 sec (reference range 24–35 sec) with normal bleeding time, prothrombin time, and thrombin time on 27 November 2015 for re‐examination.

## Discussion

Acquired hemophilia A is a bleeding disorder caused by an autoantibody to factor VIII (FVIII), presenting with serious and life‐threatening bleeding and associated with significant morbidity and mortality. There is low incidence of AHA in population (0.2–1 million/year) but high mortality (7.9–22%) according to a recent review 5. Spontaneous bleeding such as gastrointestinal bleeding, muscle bleeding, subcutaneous bleeding, urogenital bleeding, or iatrogenic bleeding after invasive examination might be found in patients with AHA 6. There were large subcutaneous bruises in this patient without history of bleeding or hemophilia‐related diseases. She had prolonged aPTT with low FVIII activity and higher FVIII inhibitor titer, and no evidence of heparin effect, clotting factor deficiency, antiphospholipid antibody syndrome, acquired hemophilia A, and von Willebrand disease (vWD). This patient was diagnosed as AHA according to the diagnostic criteria.

This patient did not have causes of AHA such as autoimmune diseases, malignancy, dermatological disorders 2, 3, 4, 5, 6. So we thought there might be some drugs leading to AHA. There were case reports about drug‐related AHA such as ivabradine 7, penicillin, ampicillin, methyldopa^8^, and clopidogrel^9^. However, this patient had clopidogrel for 1 year, and low‐molecular weight heparin was used before without abnormality of coagulation function. In addition, present laboratory tests showed that there was no abnormity in heparin contamination measured by thromboelastogram. This patient was treated by aspirin and clopidogrel after hospital discharge, and following coagulation tests were normal. So we thought there might be no relationship between AHA and clopidogrel in this patient. The level of aPTT was changed before and after Ginkgo‐dipyridamolum. So Ginkgo‐dipyridamolum might be related with AHA in this patient.

Ginkgo‐dipyridamolum was compound preparation of Ginkgo and dipyridamolum. Ginkgo biloba extract is one of the few Chinese herbal preparations that is recognized by the international medical community 10. Studies have shown that patients with type 2 diabetes might benefit from ingesting Ginkgo biloba extract to improve platelet function 11, and reduce malondialdehyde levels in platelets 12. Dipyridamolum was one kind of antiplatelet aggregation drugs which was used in the treatment of thromboembolic disease. So Ginkgo‐dipyridamolum was used in the treatment of coronary artery disease and thromboembolic disease. There was little adverse effect except for nausea, vomiting, dizziness, and skin allergy in the previous usage of Ginkgo‐dipyridamolum. However, drug‐induced AHA usually occurs several weeks to months after causative agent 9. But it took just one day after Ginkgo‐dipyridamolum usage in this case. As above showed, AHA is an autoimmune‐related disease, so we thought compound preparation of Ginkgo‐dipyridamolum especially Ginkgo might trigger a shift of immune dysfunction. That was to say, the herbal preparation might cause immune dysfunction in this patient. However, we could not explain the short interval between the intake of Ginkgo‐dipyridamolum and the development of AHA at present, and we could not get the direct experimental evidence showing the causal relationship between usage of Ginkgo‐dipyridamolum and AHA. More studies should be investigated in future.

Bleeding control and inhibitor‐removing therapy should be started as soon as possible after AHA is confirmed 13. Corticosteroids alone or in combination with cyclophosphamide can eradicate the inhibitor 14. This patient was treated with corticosteroids and cyclophosphamide immediately after confirmed diagnosis and respond well with normal aPTT, FVIII activity, and FVIII inhibitor in 6 weeks.

As far as we know, this is the first idiosyncratic acquired hemophilia that might be related with Ginkgo‐dipyridamolum especially Ginkgo and it was successfully treated with hemostasis and immune‐suppression therapy.

## Conflict of Interest

No conflict of interests, financial or otherwise, are declared by the authors.
